# Decomposition of beech (*Fagus sylvatica*) and pine (*Pinus nigra*) litter along an Alpine elevation gradient: Decay and nutrient release

**DOI:** 10.1016/j.geoderma.2015.03.024

**Published:** 2015-08

**Authors:** Torsten W. Berger, Olivier Duboc, Ika Djukic, Michael Tatzber, Martin H. Gerzabek, Franz Zehetner

**Affiliations:** a Department of Forest- and Soil Sciences, Institute of Forest Ecology, University of Natural Resources and Live Sciences (BOKU), Peter Jordan-Straße 82, 1190 Vienna, Austria; b Department of Forest- and Soil Sciences, Institute of Soil Research, University of Natural Resources and Live Sciences (BOKU), Peter Jordan-Straße 82, 1190 Vienna, Austria

**Keywords:** Climosequence, Decomposition, Elevation gradient, *Fagus sylvatica*, Litterbag, *Pinus nigra*

## Abstract

Litter decomposition is an important process for cycling of nutrients in terrestrial ecosystems. The objective of this study was to evaluate direct and indirect effects of climate on litter decomposition along an altitudinal gradient in a temperate Alpine region. Foliar litter of European beech (*Fagus sylvatica*) and Black pine (*Pinus nigra*) was incubated in litterbags during two years in the Hochschwab massif of the Northern Limestone Alps of Austria. Eight incubation sites were selected following an altitudinal/climatic transect from 1900 to 900 m asl. The average remaining mass after two years of decomposition amounted to 54% (beech) and 50% (pine). Net release of N, P, Na, Al, Fe and Mn was higher in pine than in beech litter due to high immobilization (retention) rates of beech litter. However, pine litter retained more Ca than beech litter. Altitude retarded decay (mass loss and associated C release) in beech litter during the first year only but had a longer lasting effect on decaying pine litter. Altitude comprises a suite of highly auto-correlated characteristics (climate, vegetation, litter, soil chemistry, soil microbiology, snow cover) that influence litter decomposition. Hence, decay and nutrient release of incubated litter is difficult to predict by altitude, except during the early stage of decomposition, which seemed to be controlled by climate. Reciprocal litter transplant along the elevation gradient yielded even relatively higher decay of pine litter on beech forest sites after a two-year adaptation period of the microbial community.

## Highlights

• Mass loss is higher for pine than for beech litter.
• Altitude retards decay of litter with a longer lasting effect on pine litter.
• Nutrient release is predictable by altitude during the early stage of decomposition.
• Net release of N, P, Na, Al, Fe and Mn is higher in pine than in beech litter.
• Reciprocal litter transplant yields higher decay of pine litter on beech forest sites.

## 1 Introduction

High-mountain ecosystems are especially vulnerable to climate change, since these areas will experience stronger temperature fluctuations than the global climate (Schröter et al., 2005). Mountain regions cover about one fifth of the earth's continental area but their ecological and economical importance, e.g. regarding water cycle regulation, reaches far beyond their boundaries (Beniston et al., 1997), and their soil organic carbon stocks are among the highest in terrestrial biomes (Djukic et al., 2010b; Ward et al., 2014).

Hence, research on decomposition processes, their relation to soil properties and their regulating factors in alpine ecosystems is important. Decomposition processes are important for cycling of nutrients in terrestrial ecosystems and are influenced by macro- and micro-climate, litter quality, activity of decomposing organisms and soil nutrient status (Berger and Berger, 2012, 2014; Berger et al., 2010; Coûteaux et al., 1995; Gavazov, 2010; Prescott, 2010; Vesterdal, 1999). Manipulation experiments such as soil warming (Melillo et al., 2002; Schindlbacher et al., 2011) have provided useful information on short-term soil responses to changed climatic conditions. However, such experiments offer little insight into responses that occur over longer periods, such as migration of vegetation zones. Climate-gradient studies help filling this gap via “space-for-climate” substitution. Along such gradients (climosequences), the combined effects of several factors that change with climate, can be studied, since climate change is not a mere change in temperature. Direct and indirect effects of climate may change i) soil water regimes, i.e. waterlogging or surface drying (Davidson and Janssens, 2006; Gavazov, 2010; Sjögersten and Wookey, 2004), ii) soil insulation through snow cover (Hobbie et al., 2000; Williams et al., 1998), and iii) climate driven shifts in species composition and associated litter quality (Cornelissen et al., 2007; Cornwell et al., 2008; Theurillat and Guisan, 2001). It is the combination of all these factors that will govern litter decomposition under changing climatic conditions.

Increasing elevation can select for plant species and functional groups possessing functional traits that are better adapted to nutrient limitation (Vitousek et al., 1988). The relative biomass or abundance of dominant functional groups of soil organisms can be highly responsive to elevation. For instance, the ratio of fungal to bacterial biomass can increase with elevation (Sundqvist et al., 2013; Wagai et al., 2011). Belowground consumers are intrinsically linked to aboveground communities (Wardle, 2002). Hence, changes in vegetation can shape the responses of soil communities to elevation. In a recent comprehensive review Sundqvist et al. (2013) concluded that decomposer organism densities and community composition often respond to elevation, but only few studies have explicitly tested the consequence of this fact for litter decomposition rates.

That is why, we performed a study on litter decomposition, based on a climosequence approach in the Hochschwab massif of the Northern Limestone Alps. Foliar litter of European beech (*Fagus sylvatica*) and Black pine (*Pinus nigra*) was incubated at eight sites along an elevation gradient of 1000 m (6 different altitudes at 200 m intervals) over a two-year period. Vegetation changed from alpine grassland (1900 m asl) over shrubland with mountain pine (*Pinus mugo*) bushes towards spruce (*Picea abies*) stands and finally montane beech (*F. sylvatica*) forests (900 m asl), characterized by specific climatic conditions and microbial community compositions. Previous work along this elevational gradient by Djukic et al. (2010a,b, 2012) and Duboc et al. (2012) demonstrated that differences in soil organic matter stocks and characteristics were more closely related to vegetation composition, their C input and litter quality than to variations in climatic conditions along the elevation gradient. The highest amounts of soil microbial biomass were found at sites with high soil pHs and low C/N ratios and the bacterial to fungal biomass ratio increased significantly from forest sites to shrubland and grassland sites.

The overall objective of this study was to evaluate direct and indirect effects of climate on decomposition of beech and pine litters. Below we have developed our objectives into four specific research questions accompanied by corresponding hypotheses.

1) How do beech and pine litter differ in mass loss and nutrient release during the first two years of decomposition? We used litter of beech (low-elevation sites) and pine (high-elevation sites) to test differences between these species, since it is commonly believed, that broadleaf litter decomposes faster than needle litter (Prescott et al., 2004). We hypothesized that mass loss and nutrient release are higher for beech than for pine litter.
2) Are mass loss and nutrient release more closely related to the litter type or the site of incubation (forest type), and do litter of beech and pine decay faster in their respective home environments? Using relatively (so-called) high (beech) and low (pine) quality litter enabled testing home-field advantages (HFAs) via reciprocal litter transplants at the pine and beech sites: Decomposer communities are often adapted to degrade the type of leaf litter that they encounter, which typically comes from the plant species above them, resulting in litter decomposing more rapidly in its “home” environment than in an “away” environment (Ayres et al., 2009; Veen et al., 2015; Wallenstein et al., 2013). However, several studies do not support the idea of increased decomposition of litter in its home environment, as outlined in a recent review on plant litter–decomposer affinity effects by Austin et al. (2014). Recent analyses suggest that the innate ability, or functional breadth, of the microbial community may overestimate or obscure HFA effects (Keiser et al., 2013). If litter quality is the sole driver, then more recalcitrant litter (e.g., pine; higher C/N ratio and lignin content) will decompose more slowly with any soil microbial community, regardless of its origin. On the other hand, soil microbial community may modify litter decomposition, whereby low-nutrient ecosystems have high microbial functional breadth in response to the diversity of compounds found in chemically-complex litter (the opposite is true for nutrient-rich ecosystems). Hence, in accordance with Keiser et al. (2013) we hypothesized that the soil microbial community at the pine sites (higher elevation, nutrient poor) will decompose high- (beech) and low quality (pine) litter at similar rates and the microbial community at the beech sites (lower elevation, nutrient rich) will decompose pine litter at slower rates.
3) Does altitude affect mass loss and nutrient release? Temperature is a well-known direct driver of litter decomposition, and litter mass loss and nutrient release are often greater at lower elevations due to warmer conditions (Sundqvist et al., 2013). Hence, we hypothesized that mass loss and nutrient release of decomposing litter will decline with increasing altitude, though indirect effects of climate changes along an elevation gradient (e.g., shifts in species composition and associated litter quality; see above) may obscure direct climate effects.
4) Which altitudinal changes in the environment (climate, vegetation, litter, soil, microbial community) are most closely associated with the observed decomposition patterns? Berg and McClaugherty (2008) concluded that climate is important during early stages but the later phase of litter decomposition appears to be strongly influenced by litter chemistry. We must be cautious when generalizing such statements, since responses to elevation are commonly driven by changes in temperature, and many community- and ecosystem-level variables (Sundqvist et al., 2013). Hence, given this complexity, our capacity to predict responses to elevational gradients is often limited. Nevertheless, we hypothesized that the use of simple regression techniques will contribute to disentangling direct and indirect effects of climate on litter decomposition.

## 2 Material and methods

### 2.1 Study sites

The study area is located in the Hochschwab massif of the Northern Limestone Alps. Eight sites were selected for this study along an elevation gradient from 1900 to 900 m (6 different altitudes at 200 m intervals). Site characteristics are given in Table 1. There are two sites at 1300 m (sites 1302 and 1301) and at 900 m (sites 902 and 901), in all other cases one site per altitude (sites 1900, 1700, 1500, 1100). The elevation gradient represents a climosequence from the alpine to the subalpine and montane climate/vegetation zones. Mean annual temperature ranges from 2.1 to 6.2 °C, mean annual precipitation from 1725 to 1178 mm and mean annual snow cover from 221 to 123 days between the altitudes 1900 to 900 m asl. The vegetation along the transect changes from alpine grasses and mountain pine (*P. mugo*) bushes above the timberline over spruce (*P. abies*) – and mixed spruce–beech – to beech (*F. sylvatica*) forests. All study sites are on calcareous parent material and exhibit similar soil type (Leptic Histosol, IUSS, 2006) and soil depth (between 20 and 30 cm).

### 2.2 Soils

Five replicate soil monoliths (area 20 × 20 cm) were collected from each of the eight sites and divided into the soil depths 0–5 and 5–10 cm. The forest litter layer (at the forested sites only) above the 0–5 cm soil depth layer was not part of this study. Fine soil, separated by sieving < 2 mm, was analyzed for total C and N contents by dry combustion and carbonate was measured gas-volumetrically. Organic C was calculated as the difference of total and carbonate C. For simplicity, organic C is abbreviated C throughout the paper. Soil pH was measured in deionized H_2_O at a soil:solution ratio of 1:10. More detailed methods of soil sampling and chemical analyses are given by Djukic et al. (2010b).

### 2.3 Litterbag experiment

We used a litterbag approach to measure decay and nutrient release. Details are given by Duboc et al. (2012), who used the identical litterbags at 6 sites within their study on molecular characteristics of litter materials during different decompositional stages. Shed leaves of European beech (*F. sylvatica*) and needles of Black pine (*P. nigra*) were collected in November 2006. The litter was dried at 50 °C for 24 h whereby only whole, intact leaves/needles were used, and subsequently stored at room temperature. The litterbags were made of polyethylene nets (1 mm mesh size). Bands of 40 cm × 10 cm were folded in order to obtain 10 cm × 10 cm bags, resulting in double mesh layers and closed with copper clips. Twenty bags were prepared for each site (5 replicates per litter type and 2 sampling campaigns). Each bag was filled with either 1.5 g of beech leaves or 3 g of pine needles (initial mass was recorded for each bag). In June 2007, the litterbags were placed in the soil at 5 cm depth after having been connected together with a fishing line, itself attached to a wooden pole fixed in the soil. For minimizing soil disturbance while placing the bags, a spade was used to make a narrow slit. The distance between bags was about 25 cm. At each of the two sampling campaigns in June 2008 and 2009, after one and two years of decomposition respectively, 10 litterbags (5 beeches + 5 pines) were retrieved at each site. The total number of incubated bags amounted to 160 (8 incubation sites × 2 litter mixtures × 5 replications per site × 2 sampling dates = 160). The litterbags at 1100 could not be retrieved in the second year. Soil particles were carefully removed with a brush so that only material which could be identified as litter was used for further analysis. The cleaned litter samples were oven-dried overnight at 105 °C before weighing. In order to correct the initial sample masses for the weight difference between 50 °C and 105 °C, five replicate samples of the original litter were oven-dried first at 50 °C then at 105 °C. This resulted in mean correction factors of 0.953 ± 0.001 for beech and 0.957 ± 0.0009 for pine. The samples were then individually ground through an 80 μm sieve using a Retsch ZM 1000 Ultra Centrifugal Mill.

Carbon and N contents of each litter sample were measured by dry combustion in a Carlo Erba CNS analyzer (USA) according to Tabatabai and Bremner (1991). Phosphorus, S, Ca, Mg, K, Na, Al, Fe and Mn were measured as total contents after digestion with HNO_3_/HClO_4_ (according to ÖNORM L1085) by ICP–OES (inductive coupled plasma optical emission spectrometry, Optima 3000 XL, Perkin Elmer, USA).

FT-MIR spectroscopy was applied to analyze the molecular characteristics in the same litter by Duboc et al. (2012). Spectra were obtained using a Brucker Tensor 27. Twelve bands between 3050 and 1160 cm^− 1^ were evaluated in this complimentary study. However, in this paper, we focus only on the band 1515 cm^− 1^ (starting and endpoint of integration: 1529–1494 cm^− 1^), which turned out to be a good indicator of lignin in accordance with Smidt and Meissl (2007), Smidt et al. (2008) and Nault et al. (2009). Our so-called lignin_proxy_ results from FT-MIR are semi-quantitative since the data were not calibrated against a direct measurement. Integrated areas were related to organic carbon (C), hence, lignin_proxy_ contents were given in A cm^− 1^ (mg C)^− 1^.

### 2.4 Data evaluation and statistics

Mass loss was calculated as the difference between the initial dry mass and the actual dry mass at each sampling date. Nutrient release was estimated from the initial content minus the content at each sampling date and expressed either in % of the initial content (nutrient release = 100 − remaining nutrient content in %) or in mg g^− 1^ incubated litter. The term “decomposition”, used in this study, comprises both mass loss (decay rate) and nutrient release, which are not necessarily linked with each other.

One-way ANOVAs were performed to test differences of analyzed soil parameters between the sites along the elevation gradient and to test differences of nutrient contents between litter species and years. A three-way ANOVA table of *F*-values was calculated for comparing the impact of the three factors litter species, site (altitude) and time of sampling on remaining mass, element contents and selected litter compound ratios (possible interactions between these factors indicate that that these factors affect the dependent parameter jointly). Finally, results were presented for the end of the experiment after two years (two-way ANOVA: factors litter species and incubation sites). In all cases, more than two groups were examined, Duncan multiple range tests were used to compare the associated group means.

The reciprocal litter transplant experiments at the beech (901 and 902) and mountain pine sites (1500 and 1700) were used for testing HFA. The mountain pine (*P. mugo*) sites are considered “home” for the incubated black pine (*P. nigra*) litter, since these pine species are closely related to each other assuming similar decomposition patterns. For pairwise comparisons of tree species, the HFA index gives the percentage of a more rapid (positive value) or slower (negative value) mass loss of litter when it decomposes under the tree species from which it had been derived (i.e., “at home”). We used mass loss means of the two beech (site 902 is considered a pure beech stand, though admixture of spruce amounts to 30%) and the two pine sites, respectively, for these pairwise comparisons.

To address question 4 (disentangling direct and indirect effects of climate on litter decomposition), we first performed bivariate correlations between site means of remaining mass and element contents of exposed beech and pine litters and altitude and selected soil parameters for each year of the study. In a second step, those pre-selected parameters which correlated significantly with remaining mass and element contents were used to run stepwise regressions to find the driving forces (independent variables) of decay and nutrient release (at each step, the independent variable not in the equation that has the smallest probability of *F* is entered if that probability is sufficiently small; the method terminates when no more variables are eligible for inclusion or removal). Stepwise regression is a method of data reduction, taking inter-correlations into account. Additionally, partial correlations were performed between remaining mass and element contents and altitude, being controlled for each of the measured eight soil variables (pH, C, N and C/N ratio in 0–5 and 5–10 cm soil depth) separately, and the result with the highest coefficient was given.

Effects of initial litter quality on nutrient release can be tested via linear regressions. However, since we used the same beech or pine litter and consequently the same initial litter qualities for all sites, we regressed net nutrient release (mg g^− 1^ litter; dependent variable) during the second year of the study against nutrient contents after one year (mg g^− 1^, independent variable; *N* = 7 incubation sites, without site 1100 × 5 replications per site = 35) for each element and litter species.

Stepwise discriminant analyses based on remaining element contents of C, N, S, P, S, Ca, Mg, K, Na, Al, Fe and Mn (% of initial values; remaining content = 100 − release) of beech and pine litters after one (B1, P1) and two (B2, P2) years of decomposition were performed. Remaining mass (%) was excluded, because it is used for calculating remaining nutrient contents and, thus, implicitly integrated in the used nutrient data. Grouping variables were either B1, P1, B2 and P2 (all sites) or the incubation sites, separated by litter species and year, and plotted against the first two discrimination functions. The variable selection method for stepwise discriminant analysis that chooses variables for entry into the equation was on the basis of how much they lower Wilks' lambda: at each step, the variable that minimizes the overall Wilks' lambda is entered. The strongest factors of each function, most useful for classifying between the groups, were ranked by given standardized coefficients. All statistics were performed with the package IBM SPSS Statistics 21.

## 3 Results

### 3.1 Soils

Since properties such as soil type, soil depth, slope and aspect were similar along the transect we assumed differences of soil parameters to be mainly caused by climate (altitude) and vegetation type. However, not any of the measured mean soil parameters of the eight study sites (Table 1) correlated with the corresponding altitude. Though the soils were formed on calcareous parent material, only the deepest soil layers were influenced by carbonates from the bedrock, except for site 1302, where 2.1% carbonate content was measured in 0–5 cm soil depth (the highest carbonate content of all other sites and depths amounted to 0.43%; Djukic et al., 2010b, 2013).

Regarding soil pH, two groups can be distinguished among the eight study sites: the alpine grassland site 1900, the spruce forest site 1302 and the beech forest site 1100 with pHs (0–5 cm) between 5.7 and 6.1, while the remaining five sites were characterized by soil pHs (0–5 cm) between 4.0 and 4.5.

Due to the high organic carbon contents between 272 and 481 mg g^− 1^ (Table 1), the soil horizons 0–5 and 5–10 cm were not classified as mineral soil horizons (organic carbon < 17%) but as Oa horizons (very dark layer of well decomposed humus; Soil Survey Staff, 2004). Mean soil pH was negatively correlated with mean soil C content (0–5 cm: *R* = − 0.95^⁎⁎⁎^, 5–10 cm: *R* = − 0.93^⁎⁎^; *N* = 8 sites), and the mean C/N ratio (0–5 cm: *R* = − 0.87^⁎⁎^, 5–10 cm: *R* = − 0.90^⁎⁎^; *N* = 8) but there was no relation between mean soil pH and mean soil N content (in 0–5 and 5–10 cm depth, respectively). Mean soil N contents at 0–5 cm depth were within a small range (18.0–20.5 mg g^− 1^). There was a general trend that ranges between minimum and maximum site means of the studied soil parameter were increasing from 0–5 to 5–10 cm soil depth.

### 3.2 Litter quality

Higher initial contents of N, S, Ca, Mg, K and N/lignin_proxy_ ratios and lower values for C, Fe and ratios of C/N and C/P in beech than in pine litter indicated clearly higher quality litter of the deciduous species (Table 2, Supplementary Fig. 1). Increasing nutrient contents over time reflected microbial immobilization and decreasing contents indicated nutrient release. After two years of decomposition, significant differences between beech and pine litters were recorded for all elements (except K), and ratios (year 2), still stating higher litter quality for beech than pine.

### 3.3 Mass loss

#### 3.3.1 Beech versus pine litter

The average remaining mass after two years of decomposition amounted to 54% (beech) and 50% (pine), showing a slightly (but significantly) slower decay of the broadleaf litter (Table 3). After one year of decomposition the remaining masses were 65.3 and 64.6% for beech and pine, respectively (no significant differences), indicating a rapid decline of decay rate.

#### 3.3.2 Litter type versus incubation site

The remaining mass of incubated litter was primarily affected by the time of exposure (year; Table 4). Additionally, mass loss was significantly affected both by incubation site and to a minor extent by litter species according to given *F*-values. Surprisingly, differences between the litter species did not vary with the time of sampling, since there was no interaction between these two factors. However, differences between sites were dependent on the time of sampling (significant interaction site × year) and, as a consequence, differences in remaining mass between beech and pine were related to both site and year (significant interaction litter × site × year).

#### 3.3.3 Altitude effect

**Fig. 1:**
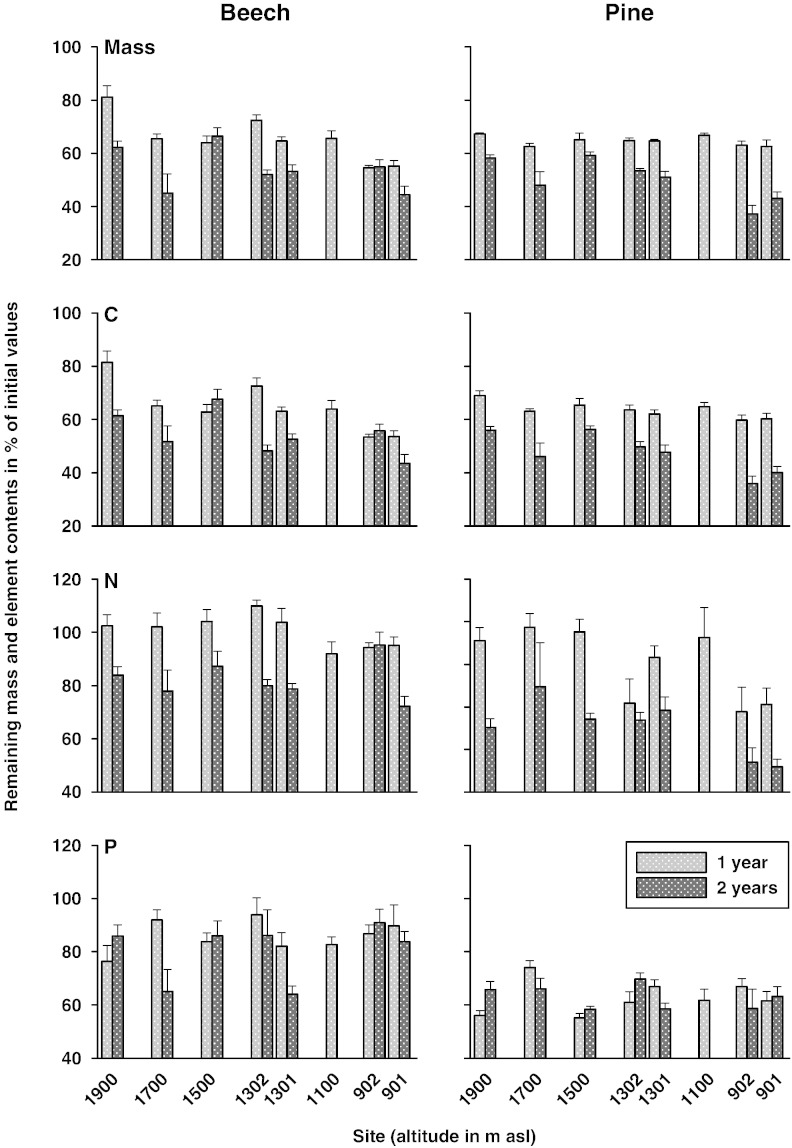
Remaining mass and contents of C, N and P (percent of initial values) of exposed beech and pine litter at the eight study sites along an elevation gradient after 1 and 2 years. Data are given as means with standard error (*N* = 5).

Site means of remaining masses after two years of decomposition, for both species jointly, are given in Table 3 and compared via multiple range tests. Excluding site 1700, the remaining masses declined consistently with decreasing altitude (three homogenous groups were separated; *a*, the lowest mean, was represented by site 901). The remaining masses of exposed beech and pine litters at the eight study sites along the elevation gradient after 1 and 2 years are plotted in Fig. 1. Altitude affected remaining mass clearly, however, within each litter species in a different year of the study: remaining mass was positively related for beech litter after 1 year (*R* = 0.80; *p* < 0.05) and for pine litter only after 2 years (*R* = 0.77; *p* < 0.05; see bivariate correlations in Table 5). There was hardly any variation of the remaining mass after one year for pine litter (coefficient of variation = 5.5%; see also Fig. 1).

### 3.4 Nutrient release

#### 3.4.1 Beech versus pine litter

**Fig. 2:**
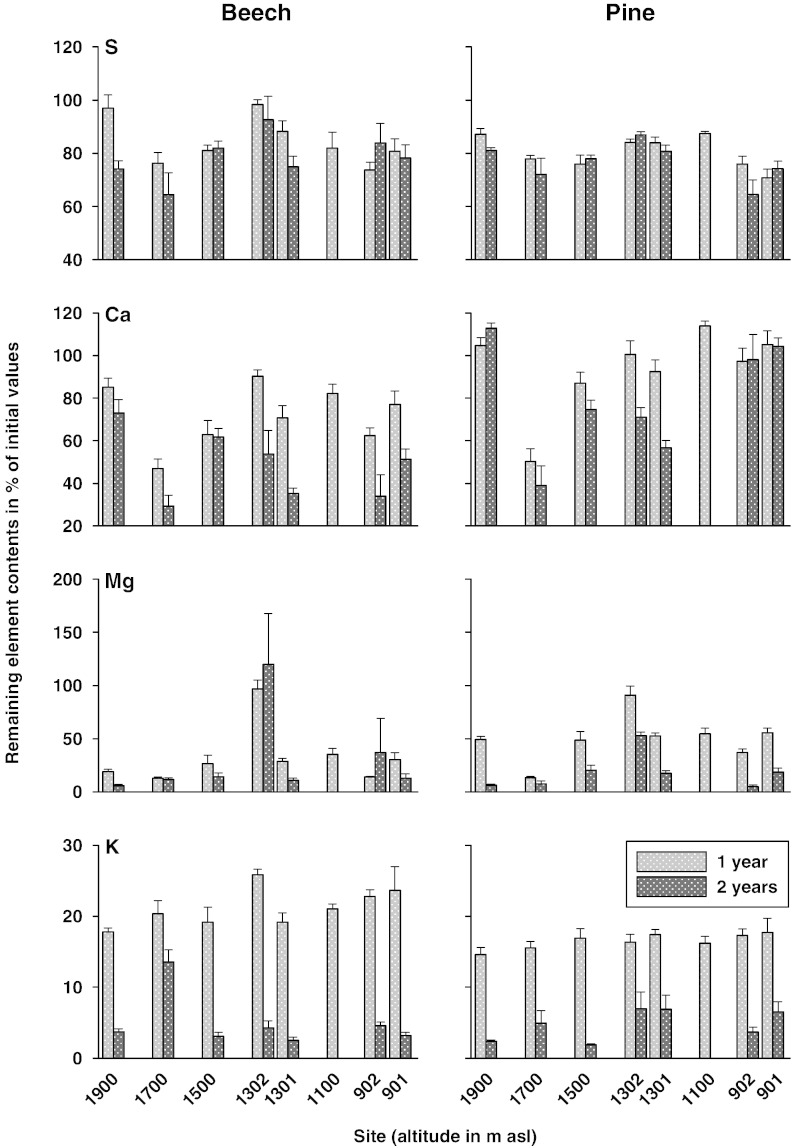
Remaining contents of S, Ca, Mg and K (percent of initial values) of exposed beech and pine litter at the eight study sites along an elevation gradient after 1 and 2 years. Data are given as means with standard error (*N* = 5).

Nutrient release was different between beech and pine in all cases except for S, Mg and K (Table 3). Average (site means) remaining carbon contents showed the same patterns as the remaining masses, indicated by positive correlation coefficients (*R* for B1: 0.99, *p* < 0.001; B2: 0.92, *p* < 0.01; P1: 0.84, *p* < 0.01; P2: 1.00, *p* < 0.001; *N* = 8 and 7 for years 1 and 2, respectively). Despite observed immobilization (retention) of S in beech and pine litters (compare Table 2), mean S contents correlated positively with mean remaining masses (*R*: 0.78–0.84 except for B2; *N* = 7–8) as well. As plotted in Figs. 1 and 2, mass loss was not related with other macronutrients, indicating that nutrient cycling is not solely determined by physical breakdown (decay).

Nutrient immobilization during the early phases of decomposition followed by release of the same nutrient during later phases was visible in beech litter for N (Fig. 1), resulting in much higher remaining N contents for beech (81%) than for pine (55%; Table 3). Temporal changes of litter nutrient contents (Table 2) indicated microbial immobilization of P in beech and pine litters, causing increasing remaining P contents from year 1 to year 2 at several incubation sites (Fig. 1) but net P release after two years was still positive in all cases.

Final remaining Ca contents were much higher in pine (80%) than in beech (49%) litter (Table 3) due to microbial immobilization (clearly indicated by values above 100% after one year at the sites 1900, 1302, 1100 and 901 and after two years at sites 1900 and 901) or other retention mechanisms in pine litter (compare increasing litter Ca contents with time in Table 2). Potassium release was the highest of all studied elements: the remaining K contents amounted to 5% (both litter species) of initial values.

#### 3.4.2 Litter type versus incubation site

Remaining element contents and selected ratios were affected by litter type (except for Mg and Lignin_proxy_) and by incubation site (except for P and Na; Table 4). Comparing *F*-values justifies the conclusion that the release of most nutrients (N, P, Ca, K, Na, Al, Fe, Mn) was more closely related to litter type. The factor incubation site had a greater influence on the release of C, S, Mg and Lignin_proxy_. However, significant interactions (litter × site or litter × site × year) indicate that these two factors jointly affected nutrient release.

#### 3.4.3 Altitude effect

Decay (mass loss and associated C release; see significant correlations above) was retarded by increasing altitude in beech litter during the first year; but within this short period the microbial decomposer community seemed to be adapted to the incubated substrate and decay was no longer correlated to altitude. In decaying pine litter, climate (altitude) had a longer lasting effect (see positive bivariate correlations for remaining mass, C and N contents after two years of incubation; Table 5). None of the other element releases was affected (slowed down; *p* < 0.05) by altitude, except release of K increased with altitude in accordance with increasing precipitation and consequently leaching out of decaying litter.

### 3.5 Relation between chemical soil parameters and decomposition

Remaining masses were negatively correlated with soil C/N ratios for both litter species during the first year (Table 5). Wide C/N ratios may stand for the fact that N-rich components are quickly decomposed and mineralized and do not necessarily point to retarded decomposition as usually cited in the literature. This conclusion is supported by the fact that, whenever remaining element contents were negatively correlated with soil N contents, either no significant (negative) correlations with soil C/N content were found or in one case (P1 for K; *p* < 0.10) a positive relation was recorded.

Remaining mass and specific element contents (B1: C, S, Ca, Al, Mn; P1: S, Al, Fe) of incubated litter were related positively to soil pH and negatively to soil C content. Since soil pH was negatively correlated with soil C content (see above) bivariate correlations are not useful for distinguishing between these two parameters. However, stepwise regressions removed soil pH (except for remaining content of Mn), indicating that soil C content (and not soil pH) was primarily related to decay and release of these nutrients. Soil N content in 5–10 cm depth (N_5–10_, Table 5) was positively related to the release of the following nutrients: for B1: N, P, Mg, Na, Fe; for B2: Mg; for P2: Mg.

In general, release of many nutrients seemed to be related to at least one of the measured eight soil variables (pH, C, N and C/N ratio in 0–5 and 5–10 cm soil depth) after the first year of the study. However, the complete lack of significant (*p* < 0.05) relations to soil characteristics after the second year, except for Mg (B2) is striking.

### 3.6 Discriminant analyses

**Fig. 3:**
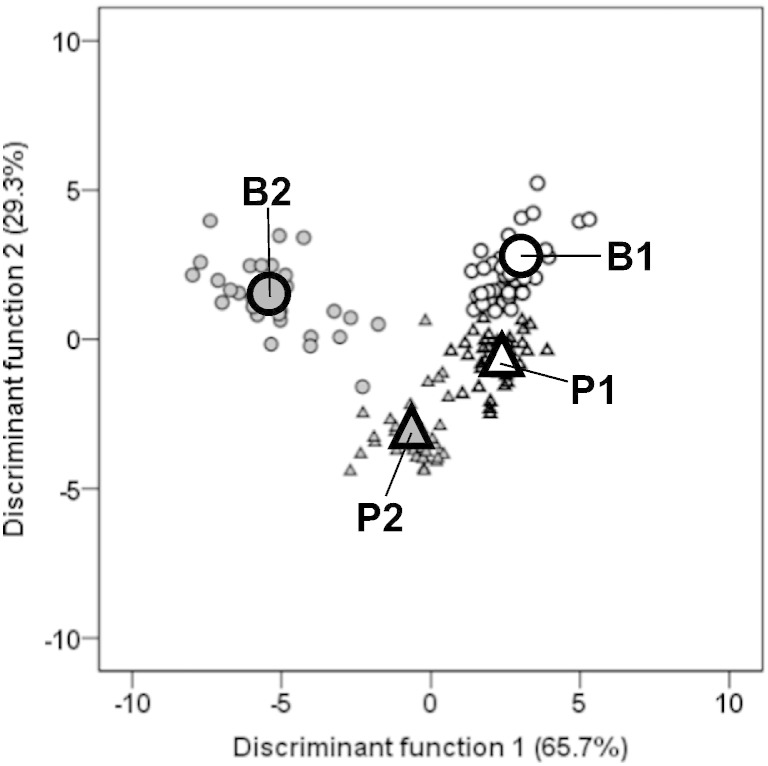
Discriminant analysis based on remaining contents of C, N, S, P, S, Ca, Mg, K, Na, Al, Fe and Mn, grouped by years of decomposition (1 year: outlined symbols; 2 years: filled symbols) and litter species (beech: circle; pine: triangle). Large symbols represent the respective group centroids: B1, B2 and P1, P2 (beech and pine after 1 and 2 years, respectively). The first two discriminant functions, ranked by percentage of explained variance (given in parenthesis), are given in Table 6.

The grouping variables B1, P1, B2 and P2 (all sites) were separated according to Fig. 3 and the strongest factors per function were remaining contents of Mn and N (Table 6). It is no surprise that the extremely high immobilization rates of Mn in beech litter during the second year (compare Tables 2 and 3) discriminated along function 1. Nitrogen was most useful for discriminating along function 2; however, effects were clearer in pine litter because of lower immobilization rates coupled with higher net-releases during the second year (see Fig. 1).

The grouping variables “incubation sites”, separated by litter species and year, are plotted in Supplementary Fig. 2. Within the first year, decomposing beech litter (B1) was hardly discriminated between the sites with the exception of 1302 and 1900, characterized by extremely high remaining contents of Mg (high remaining Mg contents in both litter species at 1302 were probably caused by the impact of measured carbonate in 0–5 cm soil depth in the form of dolomite; Fig. 2) and C (in accordance with observed relation with altitude; Table 5 and Fig. 1), respectively (compare the strongest factors of functions 1 and 2; Table 6). After two years, remaining contents of Ca and K in beech litter (B2) were the strongest factors for discriminating the sites along the functions 1 and 2. No clear patterns other than plotted in Fig. 2 were detectable (e.g., see the highest remaining K contents for site 1700).

Within the first year, decomposing pine litter (P1) was hardly discriminated between the sites with the exception of 1302 and 1700 (Supplementary Fig. 2), characterized by the highest and lowest remaining contents of the elements Mg and Ca (see Fig. 2), which contributed the most to the functions 1 and 2, respectively (Table 6). At the end of the decomposition study, pine litter (P2) caused the sites to be clearly separated along function 1 (strongest factor: Mg) between 1302 and 1900 (characterized by the lowest and highest remaining Mg contents; Table 3) and along function 2 (strongest factor: Fe) between 901 and 1700 (representing the two groups, which were separated by a multiple range test; Table 3).

## 4 Discussion

### 4.1 How do beech and pine litter differ in mass loss and nutrient release during the first two years of decomposition?

i) Mass loss of beech litter was not higher but slightly lower (*p* < 0.05) than mass loss of pine litter at the end of the study (during the first year, decay of beech and pine litters was similar). ii) Since C release was tightly related to mass loss, release of C was higher in pine litter. iii) Net release (after 2 years) of N, P, Na, Al, Fe and Mn was higher in pine than in beech litter due to high immobilization (retention) rates of beech litter. iv) However, pine litter retained more Ca than beech litter. v) Loss of K was very high and amounted to 95% of initial values for both litter species. Mean net release of S and Mg was not different between beech and pine.

Our hypothesis that mass loss is higher for beech than for pine litter was not fulfilled. Slower decay of beech versus coniferous (spruce) litter is in accordance with Vesterdal (1999; at black colour), Albers et al. (2004), Sariyildiz et al. (2005) and Berger and Berger (2012, 2014). Mass loss among five broadleaved tree species (*Acer platanoides*, *Carpinus betulus*, *F. sylvatica*, *Fraxinus excelsior* and *Tilia cordata*) was the slowest in beech litter (Jacob et al., 2010). Hence, this research has demonstrated that the purported faster decomposition of broadleaf litter than needle litter is not a safe generalization to make if beech litter is involved.

As summarized in the bullet-points above, nutrient release may be higher (according to our hypothesis), similar or lower for beech than for pine litter, depending on individual elements. As reported elsewhere (e.g., Albers et al., 2004; Prescott et al., 1993), nutrient immobilization during the early phases of decomposition may be followed by the release of the same nutrient during later phases. This kind of decomposition pattern was more pronounced in beech than in pine litter. Only Ca was immobilized in pine litter. Since Ca is very immobile and important for maintenance of cell walls (Marschner, 1986), Ca was probably retained in the slower decaying cell wall structures of coniferous litter species.

The observed net immobilization of Al and Fe in beech litter (remaining element contents above 100% in Table 3) was in accordance with Schlesinger (1997), reporting that plant litter appears to absorb Al and Fe, perhaps in compounds that are precursors to the fulvic acids. We do not know, why pine litter did not absorb Al and Fe, though Berger and Berger (2012) found high immobilization of Al and Fe in both deciduous (beech) and coniferous (spruce) litters.

For interpreting the extremely high immobilization rates of Mn in beech litter the fact that litter Mn contents increased during the decomposition study from mean values close to the analytical detection limit (0.02 mg g^− 1^) to 1.42 mg g^− 1^ (Table 2) has to be taken into consideration. Observed litter Mn contents after 2 years were in the range of initial litter Mn contents recorded by Berger and Berger (2012). The strong increase in the remaining contents of Mn may be caused by external fungal Mn (Zeller et al., 2000), since fungi dominated the microbial community at part of the studied sites (Djukic et al., 2010a).

Many element changes in decomposing litter occurred during the first year for beech and stagnated thereafter, while microbial decomposition in pine litter had a late start, especially at the low elevation sites. This finding can be seen in accordance with the general observation by Duboc et al. (2012) that molecular changes of organic compounds in decomposing litter occurred mainly in the first year for beech- but in the second year for pine litter.

### 4.2 Are mass loss and nutrient release more closely related to the litter type or the site of incubation (forest type), and do litter of beech and pine decay faster in their respective home environments?

i) Mass loss was primarily affected by incubation site, but ii) release of most nutrients was more closely related to litter type. This fact and the lack of correlations between mass loss and nutrient release (except for C and S) justifies the conclusion, in accordance with Vesterdal (1999), that nutrient cycling is not solely determined by physical breakdown (decay).

Specific plant-soil feedbacks on decomposition may influence the role of litter type versus forest type on mass loss, depending on how quickly the microbial community can adapt to the incubated litter substrate (“acclimation of decomposer communities” according to Zhou et al., 2008). Hence, we used our reciprocal litter transplant experiment at the beech (901 and 902) and mountain pine sites (1500 and 1700) for testing HFA. Calculated mean HFA was positive after one year (+ 11.4) but turned negative after two years (− 6.0; data in % faster decomposition “at home”). The positive HFA after one year was caused by the fact that at the beech sites beech litter decayed (relatively) quickly and pine litter slowly. However, at the same sites the situation turned around (relatively slow decay of beech but fast decay of pine litter) after two years, yielding a negative HFA. At the pine sites, relative mass loss was very similar for both litter species in each year, hardly effecting HFA. A negative HFA index is rare (77% of 35 reciprocal leaf litter transplants exhibited a net stimulation of decomposition “at home” with a mean HFA of + 8.0%; Ayres et al., 2009) but not unusual when beech litter is involved (Jacob et al., 2010).

Hence, in accordance with Keiser et al. (2013) our hypothesis that the soil microbial community at the pine sites (higher elevation, nutrient poor) will decompose high- (beech) and low quality (pine) litter at similar rates and the microbial community at the beech sites (lower elevation, nutrient rich) will decompose pine litter at slower rates, was supported by our data only within the first year of the study. The theory of higher microbial functional breadth in response to the diversity of compounds found in chemically-complex pine litter at high-elevation sites (therefore, the soil community does not differentiate between litter types) and of lower microbial functional breadth in response to so-called high-quality beech litter was supported by our data only on the short-term, slightly biasing (overestimating) HFA. However, in contrast to the functional breadth theory, the microbial community at the low-elevation beech sites was able to adapt quickly to the new litter substrate. The calculated negative HFA after two years is probably caused by climate — and stand mixture effects at the low-elevation beech sites. This is in accordance with Berger and Berger (2012) who concluded that increasing stand admixture of beech accelerated litter decay regardless of different quality between incubated broadleaf (beech) and needle (spruce) litter. These authors expressed increasing admixture of beech by a dummy variable for encompassing plant-induced changes in the soil environment (e.g., micro-climate, physical conditions, activity of decomposing organisms). Austin et al. (2014) pointed out that plant species may have a range of other effects on the decomposer community beyond that of litter quality that stem from rhizosphere effects (e.g., leaching or release of exudates, rhizodeposition) and symbiotic interaction with other organisms (e.g., mycorrhizas can modify the rhizosphere biota).

### 4.3 Does altitude affect decay and nutrient release?

i) Altitude retarded decay (mass loss) in beech litter during the first year and in pine litter during the second year. ii) Nutrient release declined during the first year for C, N, Na and Al in beech litter and for C, N, Al and Fe in pine litter but iii) increased for K and Ca (both litter types) with increasing altitude, if soil chemical differences between the sites were considered (controlled for). iv) During the second year, altitude did not affect nutrient release at all, except release of C and N in pine litter.

The fact that remaining contents of K (non-controlled) and Ca (controlled for soil C_0–5_) within both litter species decreased with increasing altitude during the first year of the study (Table 5, Fig. 2) is in accordance with increasing precipitation and consequently leaching out of decaying litter in accordance with high leaching rates from the green canopy (positive canopy exchange rates) as documented in numerous throughfall studies (e.g., Berger et al., 2008).

Partial correlation between remaining mass and element contents and altitude increased correlation coefficients, if controlled for one of the measured eight soil variables (the result with the highest coefficient is given in Table 5), and increased the number of significant relations in comparison to non-controlled bivariate correlations. For example, controlling for soil C content in 0–5 cm depth indicated that remaining beech litter mass and associated C increased with altitude at the highest level of significance (*p* < 0.001). Running all combinations of partial correlations after two years did not change the results of the non-controlled bivariate correlations: altitude did only control remaining mass and C and N contents in pine litter but did not affect mass loss or nutrient release in beech litter at all.

We assume that controlling for soil chemical parameters enables focusing on direct effects of altitude (climate: temperature and precipitation) by eliminating most indirect effects, since plant–soil feedbacks (e.g., Berger et al., 2004) and associated effects on the soil microbial biomass and community (Djukic et al., 2010a) are commonly reflected in the chemistry of the soil. Hence, climate controlled mass loss and release of most nutrients during early (first year) stages of decomposition in beech litter. For pine litter, climate had a longer lasting effect on mass loss and release of C and N. We conclude in accordance with Taylor et al. (1991), that physical resistance (i.e., remaining mass) was affected by climate, which lasted longer for the coniferous than the broadleaved species, influencing accessibility to decay factors.

In general, our hypothesis that mass loss and nutrient release of decomposing litter will decline with increasing altitude (though indirect effects of climate changes along an elevation gradient may obscure direct climate effects) was supported. However, the release of K and Ca increased with increasing altitude and altitude seemed to effect only early stages of decomposition. Controlling for soil chemical parameters was useful for eliminating a major part of indirect climate effects and focusing on direct climate changes with altitude.

### 4.4 Which altitudinal changes in the environment (climate, vegetation, litter, soil, microbial community) are most closely associated with the observed decomposition patterns?

i) Climate (altitude) and soil chemical parameters affected mass loss and release of most nutrients during early stages (first year) of decomposition, in which physical/chemical processes played an important role (see above). ii) Microbial decomposition after quick adaptation of the soil community to the incubated litter substrate is put forward for explaining the lack of similar correlations during later (after two years) stages. iii) Similar initial litter chemistry diverged when decomposed in different decomposer communities and – via a positive feedback loop – the influence of litter nutrient contents steadily increased during later stages of decomposition. iv) Shifting plant species compositions along the elevation gradient probably exerted the strongest influence on litter decomposition, reinforcing altitudinal trends.

The complete lack of significant (*p* < 0.05) relations between nutrient release and soil characteristics after two years (see Section 3.5), except for Mg (B2), justifies the conclusion that other parameters drive decomposition during later phases, e.g., the microbial environment (after being adapted to the newly incubated litter substrate) and the litter quality per se. Within one year of the study, the quality of the initial litter (the same beech or pine litter was used for all sites) changed (Table 2) and the ranges (maximum–minimum) of element contents in one-year-old litter increased to values high enough for regressing net nutrient release (mg g^− 1^ litter; dependent variable) during the second year of the study against nutrient contents after one year (Supplementary Table 1). The nutrient content in one-year-old litter explained a high proportion of the variation in the release of the same nutrient for all measured elements. This demonstration that similar initial litter chemistry diverged when decomposed in different decomposer communities, in accordance with Wickings et al. (2012), supports the idea of a strong interactive effect of plant litter and microbial community composition. The fact that litter quality correlated with nutrient release after the first year and, simultaneously, effects of altitude (climate) and soil chemical parameters declined, in accordance with Aerts (1997), may be interpreted as a shift from climate control of litter decay to litter chemistry control.

In general, the impact of altitude on nutrient release (especially during the second year) was small. Since not any of the measured mean soil parameters of the eight study sites correlated with altitude, we conclude that the vegetation and the litter produced created a specific environment for decomposition, biasing altitudinal trends. Several studies (Hobbie, 1996; Prescott, 2010; Wardle et al., 2009) have also suggested that climate-induced changes in rates of litter decomposition and associated feedbacks to soil fertility and productivity are likely to be small unless there is a shift in plant species composition (and thus litter quality). The outcome of the reciprocal litter transplant experiment, using selected sites along the elevation gradient, was very helpful for understanding altitudinal changes of remaining masses of beech and pine litter (see Fig. 1). The relation between beech mass loss and altitude during the first year was partly caused by the fact that beech litter decayed relatively faster at lower-lying “home-similar” sites, but ceased during the second year because decay was slowed down “at home” but not “away” (high elevation sites). On the other hand, decay of pine litter was retarded “away” (low elevation) but not “at home” (high elevation) during the first year, causing similar decay rates along the elevation gradient (compare lack of significant relation between altitude and remaining mass in Table 5). However, after adaptation of the microbial community to the decay of so-called low quality litter, pine litter decomposition was relatively sped up at low elevation sites “away”, causing a clear altitudinal trend after two years (*p* < 0.05; Table 5).

Prescott (2010) emphasized the existence of thresholds for all major driving factors of litter decomposition. E.g., correlations between temperature and decay can be found on a global scale according to Zhang et al. (2008), but if temperature is generally sub-optimal (mean annual temperature < 10 °C), decomposition is constrained and a correlation is unlikely. In the present study, the mean annual temperature is less than 10 °C and the range (2.1–6.2 °C) is narrow (Table 1). This might be another reason, why the impact of altitude on decomposition was small.

Besides shifts in plant species composition, HFA and existence of thresholds, a fourth reason why effects of altitude are commonly masked in field studies, is that micro-climatic conditions may deviate substantially from the general elevation trend. E.g., insulation by snow cover can keep the temperatures just below 0 °C and a considerable portion of the decomposition can happen during winter (Gavazov, 2010; Schindlbacher et al., 2007; Williams et al., 1998). Hence, parameters that affected snow cover thickness and/or duration (topography, exposition to wind) are put forward to explain unexpected high decay rates at the 1700 m site (see Table 3).

Finally, we conclude that altitude comprises a suite of highly auto-correlated characteristics (climate, vegetation, litter, soil chemistry, soil microbiology, snow cover) that influence litter decomposition. Hence, given this complexity, our capacity to predict responses of decay and nutrient release of incubated litter to elevational gradients is limited. Nevertheless, simple regression techniques were useful for disentangling direct and indirect effects of climate on litter decomposition as hypothesized. Direct climate influences dominated during early stages of decomposition (characterized by physical/chemical processes) but litter quality effects (characterized by microbial decomposition processes in close interaction with plant-species composition) dominated during later stages.

## 5 Conclusions

Altitude comprises a suite of highly auto-correlated characteristics (climate, vegetation, litter, soil chemistry, soil microbiology, snow cover) that influence litter decomposition. Hence, decay and nutrient release of incubated litter is difficult to predict by altitude, except during the early stage of decomposition, which seemed to be controlled by climate in our study. Mass loss of beech litter was not higher than mass loss of pine litter. Reciprocal litter transplant along the elevation gradient yielded even relatively higher decay of pine litter on beech forest sites after a two-year adaptation period of the microbial community.

## Figures and Tables

**Table 1:** Characteristics of the eight study sites along an elevation gradient from 1900 to 900 m (6 different altitudes at 200 m intervals). There are two forested sites at 1300 m (sites 1302 and 1301) and at 900 m (sites 902 and 901), in all other cases one site per altitude. The reason for these replicated sites is the fact that two transects had been established for complementary studies involving these eight sites: transect 1 (1900–1700–1500–1301–1100–902; Djukic et al., 2010a,b; Duboc et al., 2012) and transect 2 (1900–1302–901; Djukic et al., 2013). Given site characteristics are modified from these authors. Soil data are given as means with standard deviation (SD; *N* = 5) for 0–5 and 5–10 cm soil depth (Oa horizons below the litter layer).^1^.

Site	Altitude	Coordinates		Slope	Aspect from N	Vegetation	pH		C		N		C/N		M. annual	Mean	M. annual	M. annual
		WGS84					*SD*		*SD*		*SD*		*SD*		Air	Annual	Soil	Snow
							H_2_O		mg g^− 1^		mg g^− 1^		Ratio		Temperature	Precipitation	Temperature	Cover
	m asl	N	E	%	Degrees		0–5	5–10	0–5	5–10	0–5	5–10	0–5	5–10	°C	mm	°C	Days
1900	1900	47°36′07″	15°05′37″	26	200	Alpine grasses	6.0	6.1	351.4	271.5	20.5	17.8	17.2	15.2	2.1	1725	4.2	221
						Mountain pine bushes	*0.3*	*0.4*	*19.9*	*38.5*	*1.1*	*1.9*	*1.1*	*0.5*				
							*b*	*b*	*a*	*a*	*c*	*b*	*a*	*a*				
1700	1700	47°36′04″	15°05′08″	23	180	Acidophilic shrubs mountain	4.1	3.9	481.2	471.4	19.4	16.2	25.0	29.5	2.9	1616		
						Pine bushes Alpine grasses	*0.1*	*0.1*	*4.1*	*11.6*	*2.2*	*1.8*	*3.0*	*3.3*				
							*a*	*a*	*c*	*c*	*abc*	*ab*	*bc*	*e*				
1500	1500	47°35′23″	15°04′44″	16	250	Acidophilic shrubs	4.0	3.9	473.5	464.8	18.0	17.4	26.5	27.0	3.7	1506		
						Mountain pine bushes	*0.1*	*0.1*	*21.8*	*14.4*	*1.8*	*2.6*	*1.8*	*3.1*				
							*a*	*a*	*c*	*c*	*abc*	*b*	*c*	*de*				
1302	1300	47°34′27″	15°02′19″	19	225	Spruce forest	6.1	6.4	379.0	272.9	16.8	13.4	22.4	20.0	4.5	1397	5.6	162
							*0.9*	*1.1*	*60.7*	*90.7*	*2.2*	*3.2*	*1.1*	*2.2*				
							*b*	*b*	*ab*	*a*	*a*	*a*	*b*	*b*				
1301	1300	47°35′06″	15°05′15″	13	90	Mixed spruce–beech forest	4.0	3.9	446.7	443.6	17.6	16.4	25.7	27.4	4.5	1397		
							*0.1*	*0.1*	*11.0*	*19.4*	*2.4*	*2.4*	*2.9*	*3.6*				
							*a*	*a*	*c*	*bc*	*ab*	*ab*	*c*	*de*				
1100	1100	47°35′22″	15°06′01″	53	160	Beech forest	5.7	5.8	398.3	388.7	20.2	18.2	19.7	21.9	5.4	1278		
							*0.7*	*0.6*	*22.6*	*19.5*	*0.8*	*3.1*	*1.5*	*4.4*				
							*b*	*b*	*b*	*b*	*bc*	*b*	*a*	*bc*				
902	900	47°35′11″	15°06′09″	13	200	Mixed beech–spruce forest	4.5	4.5	454.7	395.3	18.0	16.4	25.5	24.1	6.2	1178		
							*0.3*	*0.3*	*24.5*	*57.1*	*2.5*	*2.0*	*2.3*	*1.3*				
							*a*	*a*	*c*	*b*	*abc*	*ab*	*c*	*cd*				
901	900	47°32′55″	15°04′03″	21	225	Beech forest	4.4	4.5	446.5	444.5	18.2	18.0	24.7	24.7	6.2	1178	6.9	123
							*0.3*	*0.3*	*9.8*	*11.4*	*1.2*	*0.3*	*1.4*	*1.1*				
							*a*	*a*	*c*	*bc*	*abc*	*b*	*bc*	*cd*				

^1^A one-way ANOVA (factor site) was performed to test differences of each soil parameter between the sites along the elevation gradient and results of a Duncan multiple range test are given (different letters in columns indicate significant differences, *p* < 0.05; *a* represent the lowest mean; *N* = 8 sites × 5 replications per site = 40).

**Table 2:** Nutrient (mg g^− 1^) and lignin proxy (semi-quantitative result expressed as absorbance A per cm^− 1^ per mg organic carbon at wavenumber 1515 cm^− 1^) contents and corresponding mass ratios of C/N, C/P and N/lignin proxy of beech and pine litter after 0 (initial values), 1 and 2 years of decomposition (means over all incubation sites).^1^.

Year	Litter	C		N		P		S		Ca		Mg		K		Na		Al		Fe		Mn		C/N ratio		C/P ratio		Lignin proxy		N/lignin proxy	
0	Beech	454.4	*a*	7.6	*a*	0.4	*a*	1.2	*a*	17.5	*a*	1.7	*a*	2.9	*c*	0.2	*a*	0.2	*a*	0.2	*a*	0.02	*a*	60.4	*b*	1172.6	*b*	0.8	*a*	9.1	*ab*
	Pine	501.9	*B*	5.8	*A*	0.4	*A*	1.0	*A*	7.2	*A*	1.2	*A*	2.4	*C*	0.1	*A*	0.2	*A*	0.3	*A*	0.02	*A*	87.7	*A*	1390.8	*B*	0.8	*A*	7.4	*B*
	*p*	***		**				***		***		***		**						^(^*^)^				***		*				^(^*^)^	
1	Beech	447.4	*a*	11.8	*b*	0.5	*b*	1.6	*b*	19.5	*a*	0.8	*a*	1.0	*b*	0.3	*b*	0.3	*a*	0.4	*a*	0.01	*a*	38.7	*a*	878.9	*a*	1.2	*b*	10.6	*b*
	Pine	493.3	*AB*	6.3	*A*	0.4	*A*	1.2	*B*	10.4	*AB*	0.9	*A*	0.6	*B*	0.1	*A*	0.2	*A*	0.2	*A*	0.03	*A*	81.3	*A*	1428.3	*B*	1.2	*B*	5.5	*A*
	*p*	***		***		***		***		***				***		***		***		***		***		***		***				***	
2	Beech	449.8	*a*	11.5	*b*	0.6	*b*	1.8	*b*	15.4	*a*	1.0	*a*	0.3	*a*	0.3	*b*	0.8	*b*	0.7	*b*	1.42	*b*	40.0	*a*	807.4	*a*	1.5	*c*	7.7	*a*
	Pine	475.7	*A*	6.5	*A*	0.5	*B*	1.5	*C*	12.0	*B*	0.4	*A*	0.2	*A*	0.1	*A*	0.2	*A*	0.3	*A*	0.05	*B*	75.9	*A*	1052.9	*A*	1.7	*B*	4.3	*A*
	*p*	***		***		***		***		*		^(^*^)^				***		***		***		***		***		***		*		***	

^1^A one-way ANOVA (factor litter species) was performed to test chemical differences between beech and pine litters for each year separately (year 0: *N* = 2 litter species × 5 replications = 10; year 1: *N* = 2 litter species × 8 incubation sites × 5 replications per site = 80; year 2: *N* = 2 litter species × 7 incubation sites × 5 replications per site = 70). Only significant results are shown as: ^(^*^)^: *p* < 0.10; *: *p* < 0.05; **: *p* < 0.01; and ***: *p* < 0.001. Another one-way ANOVA (factor year) was performed to test changes over time for each litter species separately and results of a Duncan multiple range test are given for differences in lower case letters within beech and in capital letters within pine litter (different letters indicate significant differences, *p* < 0.05; *a* and *A*, respectively, represent the lowest mean).

**Table 3:** Remaining mass and element contents (% of initial values), C/N and C/P ratios, lignin_proxy_ content (A cm^− 1^/(mg C)^− 1^) and corresponding N/lignin_proxy_ ratio in mg g^− 1^/[A cm^− 1^ (mg C)^− 1^] after two years of decomposition for the grouping factors litter species (beech, pine) and incubation site (7 sites between 1900 and 900 m asl; sites at 1300 m asl: 1302 and 1301; sites at 900 m asl: 902 and 901).^1^.

Parameter	Beech	Pine		1900		1700		1500		1302		1301		902		901	
Mass	54.3	50.0	*	60.2	*c*	46.5	*ab*	62.8	*c*	52.7	*b*	52.1	*b*	46.1	*ab*	43.7	*a*
C	54.8	47.4	***	58.7	*c*	48.5	*b*	62.0	*c*	48.9	*b*	50.1	*b*	45.8	*ab*	41.6	*a*
N	82.6	55.2	***	69.5	*b*	70.6	*b*	72.2	*b*	68.4	*b*	69.0	*b*	71.1	*b*	57.6	*a*
P	80.6	63.0	***	75.9	*bc*	65.5	*ab*	72.2	*bc*	78.0	*c*	61.4	*a*	74.8	*bc*	72.4	*bc*
S	79.1	76.8		77.6	*ab*	68.7	*a*	80.0	*b*	89.7	*c*	77.9	*ab*	74.2	*ab*	76.1	*ab*
Ca	48.8	79.6	***	93.0	*d*	34.6	*a*	68.2	*bc*	62.4	*b*	46.0	*a*	66.0	*b*	80.8	*cd*
Mg	31.5	18.5		6.1	*a*	9.6	*a*	17.3	*a*	86.4	*b*	14.6	*a*	21.1	*a*	16.1	*a*
K	4.8	4.8		3.1	*ab*	8.8	*c*	2.5	*a*	5.6	*b*	4.7	*ab*	4.1	*ab*	5.1	*ab*
Na	90.1	44.3	***	46.4	*a*	99.3	*a*	66.0	*a*	75.6	*a*	73.8	*a*	50.7	*a*	56.3	*a*
Al	277.6	48.4	***	191.6	*b*	235.3	*bc*	46.4	*a*	220.7	*bc*	53.3	*a*	310.6	*c*	56.6	*a*
Fe	217.8	40.7	***	156.1	*b*	154.6	*b*	63.6	*a*	166.5	*b*	60.9	*a*	217.3	*b*	64.2	*a*
Mn	5211.8	134.1	***	3539.0	*c*	1028.5	*a*	3538.2	*c*	2356.6	*bc*	2625.4	*bc*	2009.0	*ab*	2971.7	*bc*
C/N	40.0	75.9	***	66.4	*b*	53.7	*a*	67.0	*b*	56.3	*a*	55.4	*a*	51.2	*a*	59.0	*ab*
C/P	807.4	1052.9	***	1011.4	*cd*	945.3	*bc*	1131.1	*d*	835.0	*ab*	1040.9	*cd*	796.1	*a*	760.4	*a*
Lignin_proxy_	1.5	1.7	*	1.7	*b*	1.7	*b*	1.6	*b*	1.7	*b*	1.8	*b*	1.3	*a*	1.4	*a*
N/lignin_proxy_	7.7	4.3	***	4.9	*a*	6.0	*ab*	5.2	*a*	5.4	*a*	5.2	*a*	8.2	*c*	6.9	*bc*

^1^A two-way (2 × 7) ANOVA was performed for each parameter (*N* = 2 litter species × 7 incubation sites × 5 replications per site = 70; site 1100 with only one sampling event after 1 year could not be included in this analysis). Only significant differences between beech and pine (factor litter species) are shown as: *: *p* < 0.05; **: *p* < 0.01; and ***: *p* < 0.001. Significant results of a Duncan multiple range test are given for the grouping variable incubation site (different letters indicate significant differences, *p* < 0.05; *a* represents the lowest mean).

**Table 4:** ANOVA table of *F*-values on the effects of litter species (beech, pine), incubation site (7 sites between 1900 and 900 m asl) and year (1 and 2 years of decomposition) on the remaining mass and element contents (% of initial values), C/N and C/P ratios, lignin_proxy_ content (A cm^− 1^/(mg C)^− 1^) and corresponding N/lignin_proxy_ ratio in mg g^− 1^/[A cm^− 1^ (mg C)^− 1^] of litter enclosed in mesh bags.^1^.

Parameter	Litter(L)		Site(S)		Year(Y)		Significant interaction
Mass	6.2*		19.1***		159.4***		S × Y**, L × S × Y***
C	17.1***		21.9***		169.8***		L × Y **, S × Y ***, L × S × Y***
N	345.7***		4.5**		108.9***		L × S **, L × S × Y*
P	132.6***		2.1		3.2		L × S*, S × Y**
S	6.0*		9.8***		8.8**		S × Y*, L × S × Y*
Ca	123.4***		29.3***		53.6***		L × S***, L × Y**, S × Y***
Mg	0.3		19.3***		13.8***		L × S*, L × Y**
K	21.9***		4.7***		704.0***		L × S*, L × Y***, S × Y**, L × S × Y**
Na	44.9***		1.8		17.7***		L × S*
Al	144.9***		14.3***		17.0***		L × S***, L × Y***, S × Y***, L × S × Y***
Fe	205.9***		10.2***		5.2*		L × S***, L × Y***, S × Y***, L × S × Y**
Mn	249.4***		4.6***		264.2***		L × S**, L × Y***, S × Y***, L × S × Y***
C/N	605.9***		3.4**		1.9		L × S*, L × Y*, S × Y**
C/P	222.9***		15.8***		69.1***		L × S**, L × Y***, S × Y***
Lignin_proxy_	2.9		4.2**		77.1***		L × S*, L × Y*, S × Y**, L × S × Y*
N/lignin_proxy_	119.7***		3.4**		27.1***		L × Y*

^1^A three-way (2 × 7 × 2) ANOVA was performed for each parameter (*N* = 2 litter species × 7 incubation sites × 5 replications per site × 2 sampling dates; years = 140; site 1100 with only one sampling event after 1 year was not included in this analysis). Significant interactions between the grouping factors indicate that these factors cannot be tested individually but affect the dependent parameter jointly. Only significant results are shown as: *: *p* < 0.05; **: *p* < 0.01; and ***: *p* < 0.001.

**Table 5:** Significant bivariate correlations between mean remaining mass and element contents (C, N, P, S, Ca, Mg, K, Na, Al, Fe, Mn; % of initial values) and altitude (m asl) and soil parameters in 0–5 and 5–10 cm soil depth according to Table 1 (bold: *p* < 0.01; normal: *p* < 0.05; italic: *p* < 0.10) after 1 and 2 years of decomposition of beech and pine litters (1 year: *N* = 8; 2 years: *N* = 7, without study site 1100). In a second step, these selected variables were used to run stepwise regressions to select the driving forces (independent variables) of remaining mass and remaining element contents. Model results of these linear regression equations are shown; in case only one parameter was selected at the level *p* < 0.10, the enter method instead of the stepwise method had to be used. In addition, partial correlations were performed between each parameter (see table) and altitude, being controlled for each of the given eight soil variables separately, and the result with the highest coefficient is given (1 year: *df* = 5; 2 years: *df* = 4). Significance of adjusted coefficient of determination (*r^2^*) and partial correlation coefficients (*R*) are shown as: ^(^*^)^: *p* < 0.10; *: *p* < 0.05; **: *p* < 0.01; and ***: *p* < 0.001.

Litter years	Parameter	Stepwise regression model (enter method for 0.5 > *p* < 0.10)	Bivariate correlation coefficients									Partial correlation coefficients		
			Altitude	pH_0–5_	C_0–5_	N_0–5_	C/N_0–5_	pH_5–10_	C_5–10_	N_5–10_	C/N_5–10_	Altitude		Controlling for
Beech														
1 year	Mass	= 90.536 + 0.016 **altitude** − 0.108 **C_0–5_**; *r^2^* = 0.59 + 0.37 = 0.96***	0.80	*0.68*	− 0.74		− 0.74	*0.63*	*− 0.70*		*− 0.66*	0.97	***	C_0–5_
	C	= 90.205 + 0.017 **altitude** − 0.114 **C_0–5_**; *r^2^* = 0.61 + 0.35 = 0.96***	0.81	*0.68*	− 0.74		− 0.72	*0.63*	− 0.72		*− 0.66*	0.97	***	C_0–5_
	N	(= 145.462 − 2.689 N_5–10_; *r^2^* = 0.39 ^(^*^)^; enter method)								*− 0.69*		0.95	**	N_0–5_
	P	(= 126.403 − 2.418 N_5–10_; *r^2^* = 0.33^(^*^)^; enter method)								*− 0.65*				
	S	= 152.596 − 0.158 **C_0–5_**; *r^2^* = 0.62*		0.72	− 0.82			*0.70*	− 0.80		*− 0.71*			
	Ca	= 187.500 − 0.269 **C_0–5_**; *r^2^* = 0.73**		0.83	**− 0.88**		*− 0.67*	**0.84**	− 0.78		− 0.81	− 0.69	^(^*^)^	C_0–5_
	Mg	= 240.350 − 12.383 **N_5–10_**; *r^2^* = 0.43*								− 0.71				
	K	(= 27.517 − 0.005 altitude; *r^2^* = 0.31^(^*^)^; enter method)	*− 0.64*									− 0.81	*	N_5–10_
	Na	= 255.815 − 8.493 **N_5–10_**; *r^2^* = 0.52*								− 0.77		0.92	**	N_0–5_
	Al	= 325.754 − 0.466 **C_5–10_**; *r^2^* = 0.60*		*0.69*	*− 0.69*			*0.69*	− 0.81		*− 0.62*	0.69	^(^*^)^	pH_5–10_
	Fe	= 394.045 − 23.539 **N_5–10_** + 31.285 **pH_0–5_**; *r^2^* = 0.50 + 0.26 = 0.76*		*0.65*				*0.66*	− 0.73	− 0.76				
	Mn	= 17.721 + 9.219 **pH_5–10_**; *r^2^* = 0.62*		0.81	− 0.81			0.82	− 0.72		*− 0.70*			
2 years	Ca	(= 105.720 − 2.393 C/N_5–10_; *r^2^* = 0.44^(^*^)^; enter method)									*− 0.73*			
	Mg	= 422.324 − 23.713 N_5–10_; *r^2^* = 0.78**								**− 0.90**				
Pine														
1 year	Mass	= 74.561 − 0.428 **C/N_0–5_**; *r^2^* = 0.55*		*0.69*	− 0.76		− 0.78	*0.63*			*− 0.70*			
	C	= 66.775 + 0.005 **altitude** − 0.435 **C/N_0–5_**; *r^2^* = 0.59 + 0.20 = 0.79**	0.80				− 0.71					0.87	*	pH_5–10_
	N	= 47.871 + 0.017 **altitude**; *r^2^* = .43*	0.71									0.82	*	C_5–10_
	S	= 121.518 − 0.096 **C_0–5_**; *r^2^* = 0.47*		*0.69*	− 0.74		− 0.73	*0.63*	*− 0.62*					
	Ca	(= 160.442 − 2.805 C/N_5–10_; *r^2^* = 0.35^(^*^)^; enter method)			*− 0.64*						*− 0.67*	− 0.81	*	C_0–5_
	Mg	(− 13.855 + 13.155 pH_5–10_; *r^2^* = 0.32^(^*^)^; enter method)						*0.64*						
	K	= 15.203 − 0.002 **altitude** + 0.159 **C/N_0–5_**; *r^2^* = 0.62 + 0.24 = 0.86**	− 0.82			*− 0.70*	0.74					− 0.90	**	pH_5–10_
	Al	= 178.388 − 4.377 **C/N_5–10_**; *r^2^* = 0.68**		0.72	− 0.76		− 0.73	0.71	**− 0.85**		**− 0.85**	0.72	^(^*^)^	C/N_5–10_
	Fe	= 131.564 − 0.195 **C_5–10_**; *r^2^* = 0.72**		0.75	− 0.80		*− 0.67*	0.73	**− 0.87**		− 0.81	0.86	*	C_5–10_
2 years	Mass	= 28.231 + 0.016 **altitude**; *r^2^* = 0.50*	0.77									0.87	*	N_0–5_
	C	= 25.307 + 0.016 **altitude**; *r^2^* = 0.59*	0.81									0.88	*	N_0–5_
	N	(= 37.484 + 0.013 altitude; *r^2^* = 0.45^(^*^)^; enter method)	*0.74*									0.89	*	C/N_0–5_
	P	(= 48.083 + 3.138 pH_5–10_; *r^2^* = 0.47^(^*^)^; enter method)		*0.73*				*0.75*						
	Ca	(= 171.215 − 3.822 C/N_5–10_; ^2^ = 0.39^(^*^)^; enter method)									*− 0.70*			
	Mg	(= 149.683 − 7.940 N_5–10_; *r^2^* = 0.47^(^*^)^; enter method)				*− 0.70*				*− 0.75*				

**Table 6:** Standardized coefficients of stepwise discriminant analyses based on remaining element contents of C, N, S, P, S, Ca, Mg, K, Na, Al, Fe and Mn (% of initial values) of beech and pine litters after one (B1, P1) and two (B2, P2) years of decomposition (remaining Na content was not selected in any of the analyses). All groups are plotted in Fig. 3. Individual groups, discriminated between 8 (1 year) or 7 (2 years) incubation sites (5 replications per site; total *N* = 150) are shown in Supplementary Fig. 2. The strongest factor of each function is marked in bold (normal: second and third strongest factor, italic: remaining factors, kept in the analysis).

Group	Discriminant function	Eigenvalue	% of variance	Canonical correlation	Level of significance	C	N	P	S	Ca	Mg	K	Al	Fe	Mn
All	1	9.84	65.7	0.95	***	*0*.*31*	*0*.*13*	*0*.*09*		0.49		0.57	*− 0*.*44*	*− 0*.*12*	**− 0**.**78**
	2	4.38	29.3	0.90	***	*− 0*.*01*	**0**.**61**	*− 0*.*07*		− 0.27		0.59	*− 0*.*24*	*0*.*39*	0.53
B1	1	14.51	72.1	0.97	***	− 0.83				− 0.68	**1**.**69**		*0*.*64*		
	2	4.43	22.0	0.90	***	**0**.**80**				0.66	*− 0*.*35*		0.52		
B2	1	7.97	44.4	0.94	***	*0*.*00*				**− 1**.**82**	1.43	*− 0*.*16*	0.81		
	2	6.92	38.6	0.93	***	*− 0*.*10*				*0*.*25*	− 0.37	**1**.**42**	− 0.94		
P1	1	5.70	58.2	0.92	***				*− 0*.*24*	0.32	**0**.**90**			0.59	
	2	2.01	20.5	0.82	***				0.42	**− 1**.**05**	0.53			*0*.*22*	
P2	1	10.38	57.4	0.96	***	0.73				0.64	**− 1**.**12**			*0*.*14*	
	2	4.06	22.4	0.90	***	0.59				− 0.63	*− 0*.*23*			**0**.**93**	
