# Supplementary material for: Decomposition of beech (Fagus sylvatica) and pine (Pinus nigra) litter along an Alpine elevation gradient: Decay and nutrient release
Source: Geoderma. 2015 Aug;251-252:92–104. doi: 10.1016/j.geoderma.2015.03.024 (PMC4418737; doi:10.1016/j.geoderma.2015.03.024)
Supplement: Supplementary file 1 — Supplementary Table 1 and Supplementary Figures 1 and 2. [file mmc1.pdf]

Supplementary Table 1 Net nutrient release (mg g^{-1} litter) of exposed beech and pine litter during the second year of the study. Results of linear regressions (including a constant) between nutrient contents after one year (mg g^{-1}; independent variable) and net nutrient release from the end of the first to the end of the second year (mg g^{-1} litter; dependent variable) are given if significant: correlation coefficient (*R*) and slope. The range (maximum – minimum) of the litter nutrient contents after one year (mg g^{-1}) defines the scale of the independent variable.[1]

Parameter	Litter	C	N	P	S	Ca	Mg	K	Na	Al	Fe	Mn
Release	Beech	61.11 **	2.17 *	0.03	0.09	6.23 ***	0.04 *	0.76 ***	0.07	-0.32 ***	-0.18 **	-1.21 ***
	Pine	123.75	1.26	0.00	0.04	1.30	0.56	0.45	0.06	0.07	0.07	-0.01
R	Beech	0.38 *		0.45 **		0.51 **		0.82 ***	0.42 *		0.51 **	-0.41 *
	Pine		0.65 ***	0.63 ***	0.36 *		0.70 ***	0.63 ***	0.41 *	0.67 ***	0.85 ***	
Slope	Beech	3.42		0.50		0.74		0.85	0.98		1.32	-63.92
	Pine		0.72	0.65	0.55		0.49	0.91	1.01	0.81	1.05	
Range	Beech	49.63	6.97	0.57	1.06	21.48	2.36	1.25	0.28	0.64	1.08	0.02
	Pine	100.02	4.80	0.20	0.38	12.43	1.86	0.66	0.19	0.33	0.49	0.07

[1]A one-way ANOVA was performed to test differences of nutrient release between beech and pine litter (N = 7 incubation sites x 5 replications per site = 35). Only significant results are shown as: *: $p < 0.05$; **: $p < 0.01$; ***: $p < 0.001$.

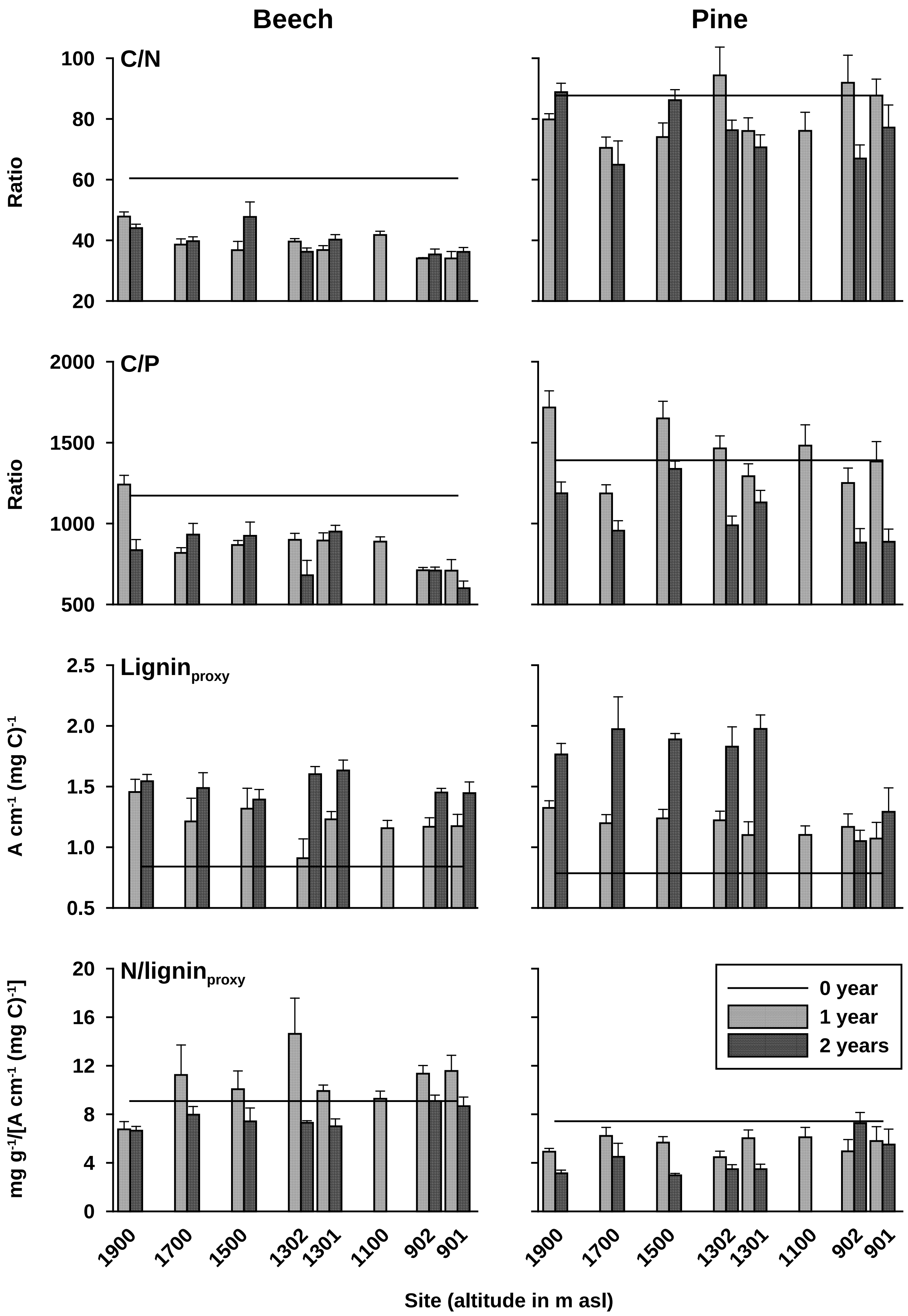

Supplementary Fig. 1. Mass ratios of C/N and C/P (non-dimensional), lignin$_{proxy}$ content (semi-quantitative result expressed as absorbance A per cm^{-1} per mg organic carbon (C) at wavenumber 1515 cm^{-1}) and the corresponding N/lignin$_{proxy}$ ratio in mg g^{-1}/[A cm^{-1} (mg C)$^{-1}$] at the eight study sites along an elevation gradient after 0 (initial values), 1 and 2 years. Data are given as means with standard error (*N* = 5).

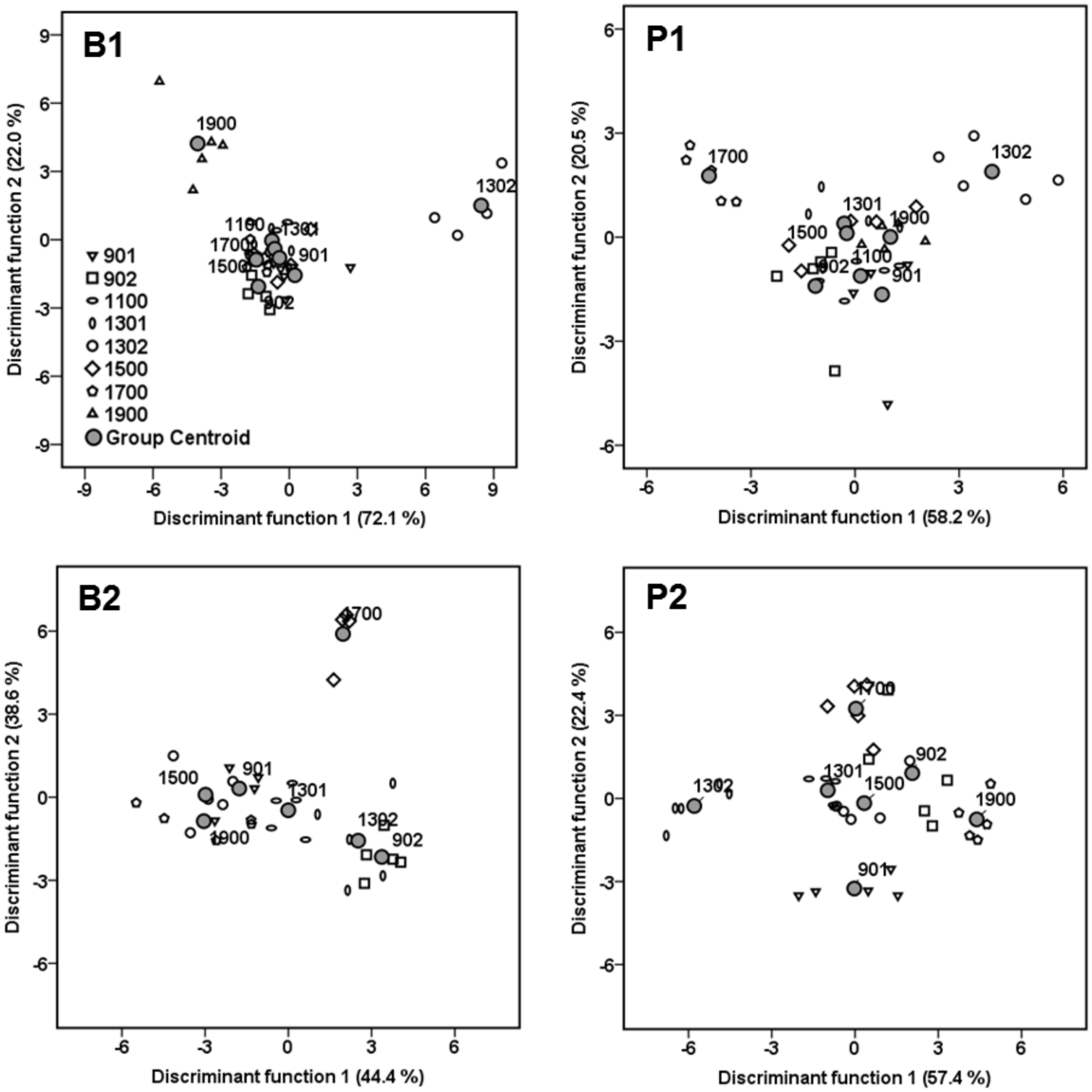

Supplementary Fig. 2. Discriminant analysis based on remaining contents of C, N, S, P, S, Ca, Mg, K, Na, Al, Fe and Mn of beech and pine after 1 year (B1, P1) and 2 years of decomposition (B2, P2), grouped by incubation site (1900 to 900 m asl). Large filled circles represent the respective group centroids of the eight sites. The first two discriminant functions, ranked by percentage of explained variance (given in parenthesis), are specific for each group and given in Table 6.
